# 976. E coli Periprosthetic Joint Infections: Poor Infection Clearance at One Year

**DOI:** 10.1093/ofid/ofac492.818

**Published:** 2022-12-15

**Authors:** Breanna A Polascik, Mikhail A Bethell, Damon V Briggs, Kwabena Adu-Kwarteng, Billy I Kim, Ayden Case, Isabel P Prado, Colleen M Wixted, Edward F Hendershot, William A Jiranek, Jessica Seidelman, Thorsten M Seyler

**Affiliations:** Duke University School of Medicine, Durham, North Carolina; Duke University, Durham, North Carolina; Duke University, Durham, North Carolina; Duke University, Durham, North Carolina; Duke University, Durham, North Carolina; Duke University, Durham, North Carolina; Duke University, Durham, North Carolina; Duke University, Durham, North Carolina; Duke University, Durham, North Carolina; Duke University, Durham, North Carolina; Duke University School of Medicine, Durham, North Carolina; Duke University, Durham, North Carolina

## Abstract

**Background:**

*Escherichia Coli* (E coli) is a gram-negative rod that can cause devastating periprosthetic joint infections (PJIs) in patients with total hip and knee replacements (THA/TKA). Minimal literature exists on outcomes of E coli PJIs.

**Methods:**

Retrospective review of our institution’s electronic medical record from 2009-2020 identified 21 patients that met MusculoSkeletal Infection Society criteria for E coli hip or knee PJI. Primary outcome was 1-year infection clearance - eradication of infection off antibiotics with no further surgeries for 1 year after completion of standard postoperative antibiotics. Minimum followup was 1 year.

**Results:**

We analyzed 21 patients (mean age 66.6 yrs, 47.6% male, 23.8% nonWhite, 38.1% knee PJIs). There were 11 acute, 8 acute hematogenous (AH), and 2 chronic PJIs. Several patients had recent gastrointestinal/urinary tract surgery (14.3%), recurrent urinary tract infections (9.5%), or >1 E coli urine culture <1 mo pre-PJI (14.3%). Surgical treatments included DAIR (66.7%), 2-stage revision (14.3%), Girdlestone/Resection Arthroplasty (G/RA; 14.3%), and fusion (4.8%), with 7.1%, 100%, 66.7%, and 100% 1-year infection clearance, respectively, and 33.3% 1-year infection clearance overall (p=.001). Common reasons for treatment failure were reinfection requiring surgery (57.1%) and chronic antibiotics (38.1%). Patients clear at 1 year had a longer mean time from most recent surgery to index PJI surgery (48.7 vs 7mo;p=.043) and more AH than acute or chronic infections (54.6% vs 27.3% vs 18.2%;p=.0412). Patients who were not clear at 1 year had more acute infections (80% vs 20% AH;p=.0412). The E coli PJI persisted in 23.8% of patients. Outcomes at final followup included G/RA (28.6%), original prosthetic (28.6%), new prosthetic (19%), above knee amputation (9.5%), destination spacer (9.5%), and arthrodesis (4.8%).

**Conclusion:**

E coli PJI 1-year infection clearance is poor, with DAIR being the most common yet least effective surgical treatment. Most E coli PJIs occurred postoperatively as opposed to hematogenously, as is sometimes assumed. This serves as a foundation for future studies evaluating E coli treatment outcomes.

**Disclosures:**

**William A. Jiranek, MD**, Biomech Holdings LLC: Stocks/Bonds|DePuy, A Johnson & Johnson Company: IP royalties **Thorsten M. Seyler, MD, PhD**, Heraeus: Paid consultant|Pattern Health: IP royalties|Restor3d: IP royalties|Smith & Nephew: Paid consultant|Total Joint Orthopedics: Paid consultant|Zimmer: research.

